# Upregulation of CENPM promotes breast carcinogenesis by altering immune infiltration

**DOI:** 10.1186/s12885-023-11808-z

**Published:** 2024-01-10

**Authors:** Yanchu Tong, Tongzhou Zhou, Xiaokun Wang, Shun Deng, Lu Qin

**Affiliations:** 1 Jingzhou Central Hospital, No. 60 Jingzhong Road, Jingzhou District, Jingzhou City, 434020 Hubei Province China; 2 The HongKong Polytechnic University, 11 Yuk Choi Road, Hung Hom, Kowloon, 999077 HKSAR China

**Keywords:** CENPM, Breast cancer, Cell proliferation, Immune infiltration, Oncogene

## Abstract

**Background:**

The involvement of centromere protein M (CENPM) in various types of cancer has been established, however, its impact on breast cancer and immune infiltration remains unknown.

**Methods:**

We examined the expression of CENPM in different cancer types by utilizing the Cancer Genome Atlas (TCGA) and Genotype Tissue Expression Pan-Cancer (GEO) databases. Using data from the TCGA, we examined the correlation between the expression of CENPM, the prognosis, and the clinicopathological features of individuals diagnosed with breast cancer. We conducted an enrichment analysis of CENPM using the clusterProfiler R software tool, utilizing data obtained from breast cancer patients and specimens at our institution. In addition to examining the correlation between CENPM expression and genes associated with immune checkpoints, the TIDE algorithm was employed to explore the potential of CENPM as a biomarker for immunotherapy in breast cancer. The impact of CENPM on the growth of breast cancer cells was evaluated through the utilization of the CCK8 test and the colony formation assay. The effect of CENPM on the migration of breast cancer cells was assessed using scratch and transwell assays.

**Results:**

Research findings indicate that elevated levels of CENPM are linked to patient outcomes in breast cancer and various clinicopathological features. Furthermore, elevated levels of CENPM expression correlated with decreased levels of CD8 + T cells and mast cells, increased levels of Tregs and Th2, and reduced levels of CD8 + T cells. Additionally, the coexpression of CENPM with the majority of genes related to immune checkpoints indicates its potential to forecast the effectiveness of treatment in breast cancer. Suppression of CENPM hampers the growth and movement of breast tumor cells.

**Conclusions:**

In summary, our study findings indicate that CENPM may serve as a cancer-causing gene in breast cancer and also as a biomarker for predicting the efficacy of immunotherapy.

The oncogene CENPM is associated with breast cancer and is involved in cell proliferation and immune infiltration.

## Background

The occurrence of cancer has risen over the past four decades. Breast cancer incidence increased by an average of 0.5% year between 2010 and 2019 [1]. Breast cancer treatments encompass various systemic approaches such as chemotherapy, endocrine therapy, targeted therapy, and immunotherapy, alongside local options like surgery and radiation therapy. Identifying patients who could potentially benefit from targeted or endocrine therapy relies heavily on conventional biomarkers such as ER, PR, and HER-2 [2, 3]. Due to the heterogeneity of tumors, the effectiveness of treatment is diminished as a result of resistance to endocrine therapies, chemotherapy, and targeted therapies [4–7]. The prognosis for breast cancer patients has significantly improved due to the promotion of early screening, the utilization of advanced technology, and increased awareness [8–10]. However, because of their unclear pathogenesis and absence of effective therapeutic targets, various pathological subtypes of breast cancer (BRCA), including triple negative breast cancer (TNBC), still necessitate enhancements in their response to treatment and prognosis [11–13].

The centromere proteins (CENPs), which form a group of proteins associated with the mitophagy-mitochondrial complex, play a crucial role in both mitophagy function and chromosome segregation during mitosis. The kinetochore protein complex, which is recruited by the mitogens, helps replicated chromosome pairs orient biologically to the meiotic spindle structure. The mouse mammary epithelium has revealed the presence of Proliferation-Associated Nuclear Element 1 (PANE1), alternatively referred to as mitogenic protein M (CENPM). Chromosome segregation during cell division is affected by it. Research suggests that CENPM has the potential to be a biomarker for predicting the development and progression of pancreatic cancer [14]. Additionally, it may function as both a biomarker and therapeutic target for hepatocellular carcinoma (HCC) due to its strong association with disease progression [14, 15]. Nevertheless, the connection between CENPM and breast cancer remains unclear.

In this study, the analysis of TCGA data was conducted to investigate the expression of CENPM in various types of cancers and its correlation with patient prognosis. In our study, we also investigated the impact of CENPM expression on the molecular pathways of breast cancer. Furthermore, we explored the correlation between CENPM expression and infiltration of immune cells, along with the pathways linked to immune checkpoint inhibitors. In the end, we conducted experimental verification to assess the effects of reducing CENPM on biological dysfunctions such as the migration and proliferation of BC cells. The results of our study suggest that CENPM could potentially function as a gene responsible for breast cancer development, in addition to serving as a promising biomarker for treatment effectiveness and a novel target for immunotherapy in breast cancer.

## Materials and methods

### Collected and processed data

Information regarding mRNA expression and clinical data of breast cancer patients was acquired from the TCGA database (*n* = 1212) and GTEx database (*n* = 179). Information regarding the survival curve was obtained from the KM plotter website (https://www.kmplot.com.).

### Collection of pathological samples

From September 2020 to February 2022, we collected 76 breast cancer specimens from Tongji Hospital, including 24 pairs of fresh frozen tissues containing cancer and paired paracancerous tissues. A total of 52 instances involved the collection of paraffin-embedded tissues, out of which 21 instances included both cancerous and paracancerous tissue samples. All experimental protocols were approved by the Ethics Committee of Tongji Hospital, in compliance with the Helsinki Declaration (approval number TJIRB20221218). Informed consent was obtained from all subjects or their legal guardians.

### Pathological sample processing

The growths and tissues adjacent to cancer were preserved in a solution of 10% formalin, embedded in paraffin, and cut into consecutive sections with a thickness of 5 μm. The slides underwent dewaxing, rehydration, and antigen extraction through microwaving. Next, the samples were placed in an incubator set at a temperature of 1 degree Celsius and treated with a diluted solution of CENPM antibody (AFFINITY, df2315) at a ratio of 1:100. Secondary antibodies were incubated for 30 min, followed by staining with DAB substrate and subsequent restamping with hematoxylin. Quantitative and statistical analysis was conducted on all immunohistochemical images using Image J and AI software.

### Analyses of correlation and enrichment

The TCGA BRCA data showed a correlation between the mRNA levels of CENPM and breast cancer. For GSEA enrichment analysis, we chose 300 genes that exhibited the highest positive correlation with CENPM to ascertain its function. Pathway analysis using GO/KEGG (http://www.genome.ad.jp/kegg/) was conducted on the 291 genes exhibiting Foldchange values above 1.5. The genes contained in the corresponding pathways were collected and analyzed by the R software GSVA package by selecting the parameter method = 'ssgsea', and finally the correlation between the genes and the pathway scores was analyzed by Spearman correlation. All the above analytical methods and R packages were performed using R software version v4.0.3. *p*< 0.05 was considered statistically significant. TIDE uses a set of gene expression markers to assess 2 distinct mechanisms of tumor immune escape, including dysfunction of tumor-infiltrating cytotoxic T-lymphocytes (CTLs) and rejection of CTLs by immunosuppressive factors. High TIDE scores are associated with poor efficacy of immune checkpoint-blocking therapy (ICB) and shorter survival after receiving ICB [16]. The graphical analysis was performed using the R (v4.0.3) package ggplot2 (v3.3.3) and ggpubr (0.4.0).

### Cell culture and treatment

SKBR3, MDA-MB-231, and MCF7 cells were cultured in DMEM medium, while MDA-MB-468 cells were cultured in RPMI-1640 medium. The STR method was employed to identify and compare all cell lines that were bought with authoritative databases. All cell lines were purchased from the Chinese Academy of Sciences Cell Bank (China).

### Extraction and quantitative RT-PCR of RNA

RNA isolation for qRT-PCR was performed using TRIzol reagent (Invitrogen, USA). Primers for CENPM and GAPDH were acquired from DynaScience Biotechnology, China. The amplification sequences were as follows: forward—GCGGACTCGATGCTCAAAGA (5'-3') CENPM, reverse—TTCTGGAGACTGTATTTGCTGTG.The forward sequence for GAPDH is GGAGCGAGATCCCTCCAAAAT, and the reverse sequence is GGCTGTTGTCATACTTCTCATGG.The qRT-PCR protocol involved 40 repetitions at a temperature of 95 °C for a duration of five minutes and 60 °C for a duration of thirty seconds, while normalizing the relative expression levels to the internal control.

### CCK8 Assay

We selected cells at the logarithmic growth stage in good condition after digesting and resuspending them in complete medium overnight. Cell proliferation was assessed using the Cell Counting Kit-8 (Invitrogen, USA) at 1d, 2d, 3d, and 4d. For the measurement of optical density at 450 nm, a Molecular Devices enzymatic digitizer was used.

### Colony-formation assay

After a 15-day period of cultivation, 3000 BC cells were placed onto plates and immobilized using 4% polyacetal. Subsequently, the cells were treated with crystal violet for staining. Cell colonies were quantified and statistically analyzed using ImageJ and artificial intelligence (AI) software.

### Transwell assay

Around 30,000 cells of breast cancer are placed in the top part of a transwell chamber. After being incubated at a temperature of 37 °C for a period of 24 h, the cells became attached to the upper part of the chamber, which was subsequently eliminated. The number of migrating cells on the bottom surface of the chamber was determined by fixing the cells with paraformaldehyde and staining them with crystal violet.

### Scratch test

Two hundred thirty-one and MCF-7 BC cells were inoculated into two-well IBIDI inserts and incubated overnight in a 24-well plate. Following the removal of the inserts from the impeccably clean table, each well was supplemented with low-serum medium, and the migration of cells was captured using a light microscope at 0 and 24 h after the removal of the inserts.

### Invasion of immune cells

We employed the GSVA package to examine the infiltration of immune cells in breast cancer. Utilizing ssGSEA data, we categorized TCGA breast cancer samples into two groups according to the medians of CENPM expression, and subsequently compared the levels of immune cell infiltration. The TIDE (Tumor Immuno Dysfunction and Exclusion) algorithm predicts whether a sample or a subtype will respond to immune checkpoint inhibitors.

### Cytokine enzyme-linked immunosorbent assays

Cytokines secreted by macrophages were measured by ELISA assay. Supernatant-treated macrophage medium from various breast cancer cell groups (NC and siCENPM) were collected. To determine the absorbance at 450 nm, an enzyme indicator was employed, and a reference graph was utilized to ascertain the value in picograms per milliliter (pg/ml).

### Immunofluorescence microscopy

The cells were subjected to 4% paraformaldehyde treatment for a duration of 15 min. Next, the actin and nuclei were stained with rhodamine ghost pen cyclic peptide at a concentration of 2.5 units/ml and DAPI, respectively. The stained cells were subsequently analyzed using fluorescence microscopy.

## Results

### Characteristics of the patient

The group included 1065 individuals diagnosed with breast cancer, possessing clinical data and RNA sequencing information. Out of these, 110 patients were paired with adjacent normal tissue samples obtained from the TCGA. We acquired gene expression information for 1799 healthy breast tissues from the GTEx database. The clinical and pathological characteristics of these patients are summarized in Table 1.

**Table 1:** Patient baseline data sheet from TCGA-BRCA

Characteristic	Low expression of CENPM	High expression of CENPM	p
n	541	542	
T stage, n (%)			< 0.001
T1	173 (16%)	104 (9.6%)	
T2	290 (26.9%)	339 (31.4%)	
T3	59 (5.5%)	80 (7.4%)	
T4	19 (1.8%)	16 (1.5%)	
N stage, n (%)			0.782
N0	256 (24.1%)	258 (24.2%)	
N1	181 (17%)	177 (16.6%)	
N2	54 (5.1%)	62 (5.8%)	
N3	41 (3.9%)	35 (3.3%)	
M stage, n (%)			1.000
M0	450 (48.8%)	452 (49%)	
M1	10 (1.1%)	10 (1.1%)	
Pathologic stage, n (%)			0.015
Stage I	109 (10.3%)	72 (6.8%)	
Stage II	295 (27.8%)	324 (30.6%)	
Stage III	112 (10.6%)	130 (12.3%)	
Stage IV	10 (0.9%)	8 (0.8%)	
Race, n (%)			< 0.001
Asian	22 (2.2%)	38 (3.8%)	
Black or African American	51 (5.1%)	130 (13.1%)	
White	434 (43.7%)	319 (32.1%)	
Age, n (%)			0.286
< = 60	291 (26.9%)	310 (28.6%)	
> 60	250 (23.1%)	232 (21.4%)	
Histological type, n (%)			< 0.001
Infiltrating Ductal Carcinoma	355 (36.3%)	417 (42.7%)	
Infiltrating Lobular Carcinoma	133 (13.6%)	72 (7.4%)	
ER status, n (%)			< 0.001
Negative	77 (7.4%)	163 (15.7%)	
Indeterminate	0 (0%)	2 (0.2%)	
Positive	439 (42.4%)	354 (34.2%)	
PR status, n (%)			< 0.001
Negative	123 (11.9%)	219 (21.2%)	
Indeterminate	2 (0.2%)	2 (0.2%)	
Positive	390 (37.7%)	298 (28.8%)	
HER2 status, n (%)			0.052
Negative	298 (41%)	260 (35.8%)	
Indeterminate	7 (1%)	5 (0.7%)	
Positive	67 (9.2%)	90 (12.4%)	
PAM50, n (%)			< 0.001
Normal	29 (2.7%)	11 (1%)	
LumA	386 (35.6%)	176 (16.3%)	
LumB	55 (5.1%)	149 (13.8%)	
Her2	29 (2.7%)	53 (4.9%)	
Basal	42 (3.9%)	153 (14.1%)	
Menopause status, n (%)			0.963
Pre	118 (12.1%)	111 (11.4%)	
Peri	21 (2.2%)	19 (2%)	
Post	357 (36.7%)	346 (35.6%)	
Anatomic neoplasm subdivisions, n (%)			1.000
Left	281 (25.9%)	282 (26%)	
Right	260 (24%)	260 (24%)	
Age, median (IQR)	59 (48, 67)	58 (49, 67)	0.573

The samples in the TCGA-BRCA database were de-duplicated, samples with missing clinical data were removed, and the samples were sorted in ascending order, and the median was used as the cut-off value to divide the samples into the CENPM low-expression group and the high-expression group.

*P* values in Table 1 are the results of the chi-square test.

### CENPM expression analysis

**Fig. 1:**
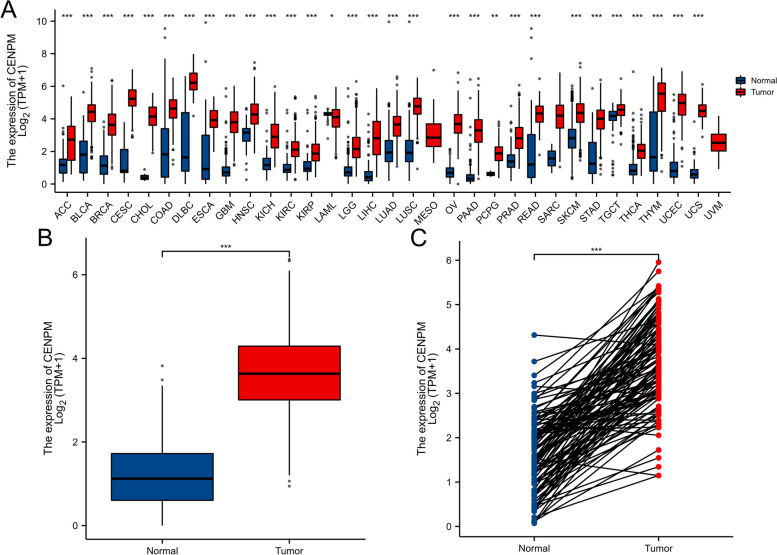
The expression difference of CENPM in cancer tissue and normal tissue.**A** Expression of CENPM in pan-cancer and adjacent normal tissues in TCGA and GTEx databases. **B** Expression of CENPM in unpaired breast cancer samples in TCGA-BRCA database. **C** Expression of CENPM in paired breast cancer samples in TCGA-BRCA database. Data were shown as mean ± SD. *p < 0.05, **p < 0.01, ***p < 0.001

To begin with, we analyzed the expression of CENPM in pancancer databases TCGA and GTEx; 29 tumors exhibited elevated levels compared to the regular tissue. The illustration depicted in Fig. 1A.

TCGA data revealed that BRCA breast cancers exhibited high expression in both unpaired (Fig. 1B) and paired (Fig. 1C) samples. The independent samples t-test and the paired samples t-test were employed as statistical approaches.

### Predicting the outlook of individuals with breast cancer by evaluating the levels of CENPM expression

**Fig. 2:**
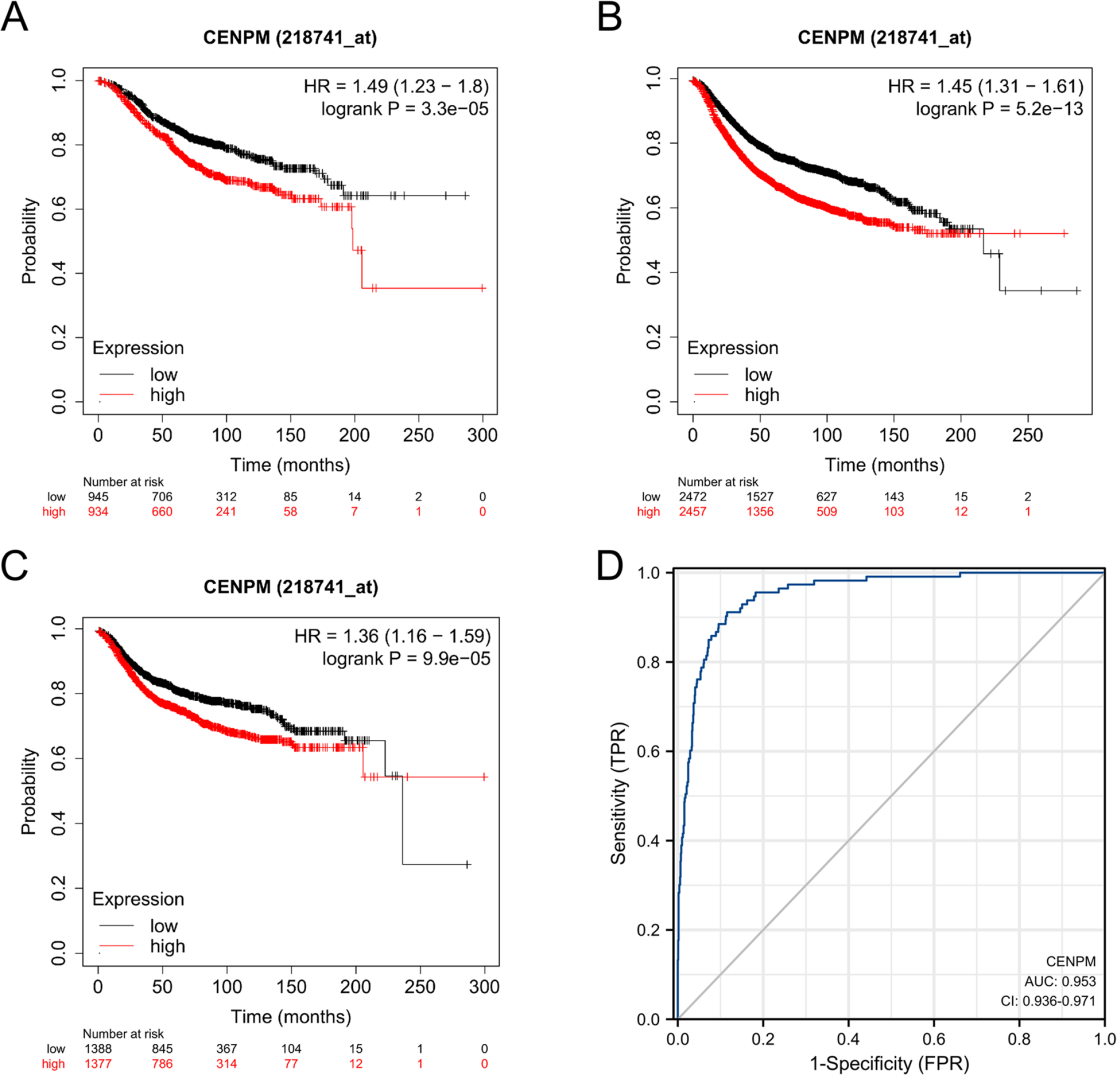
Expression of CENPM and prognosis of breast cancer patients. **A** OS of breast cancer patients based on CENPM expression level. **B** RFS of breast cancer patients based on CENPM expression level. **C** DMFS of breast cancer patients based on CENPM expression level. **D** ROC curve of CENPM

In a group of individuals diagnosed with prostate cancer, the correlation between CENPM expression and patient prognosis was assessed by examining its association with OS (Fig. 2A), RFS (Fig. 2B), and DMFS (Fig. 2C). A correlation was observed between elevated CENPM levels in breast cancer patients and a worse prognosis. In the TCGA-GTEx-BRCA database, ROC curves (Fig. 2D) were generated to assess the accuracy of diagnosing breast invasive carcinoma using CENPM expression levels (AUC = 0.953, CI 0.936–0.971). The statistical analysis revealed significant associations between CENPM expression levels and OS (1.49, *P* = 3.3e-05), RFS (1.45, *P* = 5.2e-13), and DMFS (1.36, *P* = 9.9e-05) in the study population.

### Clinically significant associations between CENPM expression and clinical variables

**Fig. 3:**
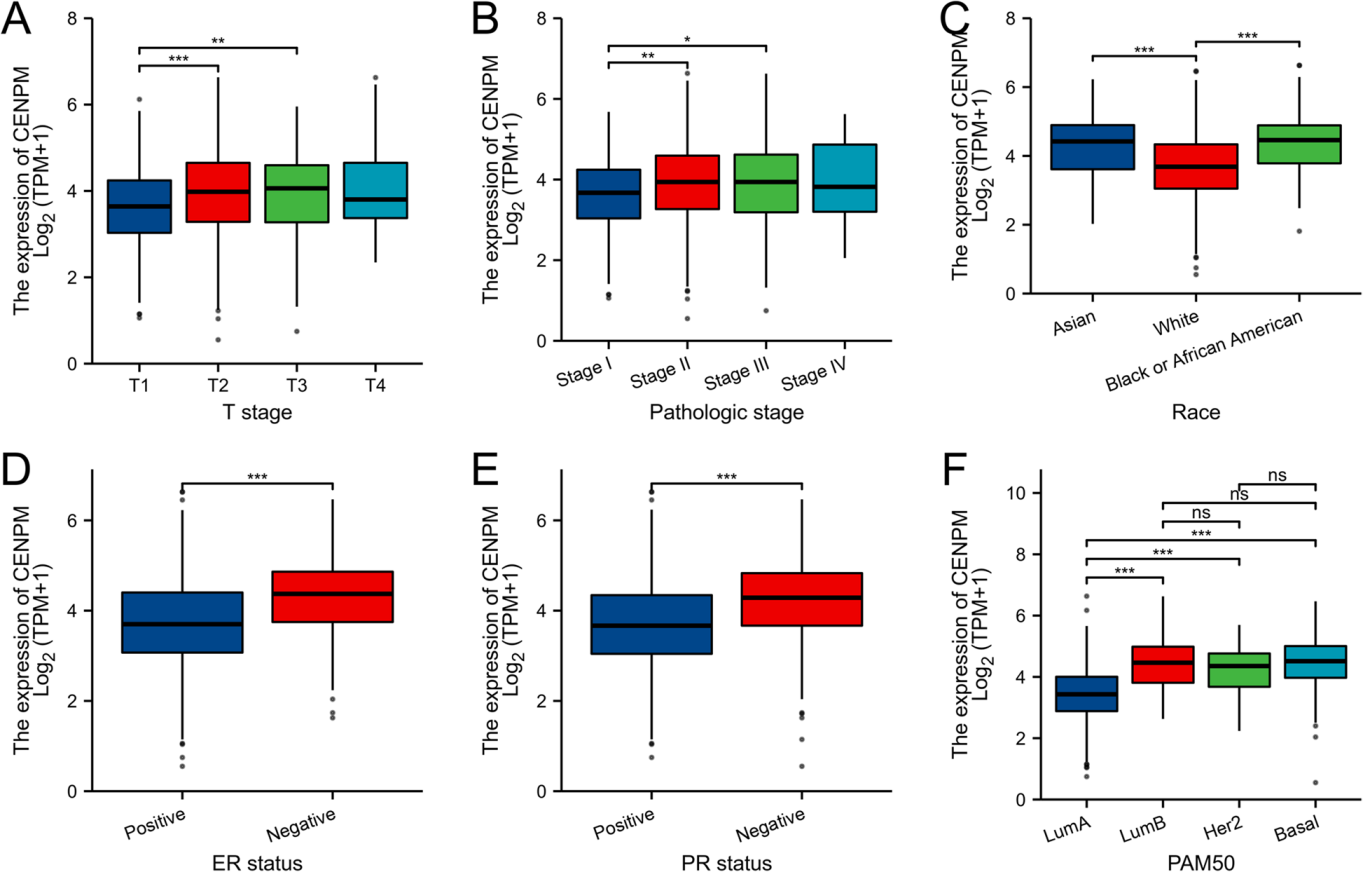
Relationship between CENPM expression and clinicopathologic features of breast cancer patients in TCGA. Data are shown for **A** T stage; **B** Pathologic stage; **C** Race; **D** ER status; **E** PR status; **F** PAM50; *p < 0.05, **p < 0.01, ***p < 0.001. ER, estrogen receptor; PR, progesterone receptor; LumA, Luminal A; LumB, Luminal B

**Fig. 4:**
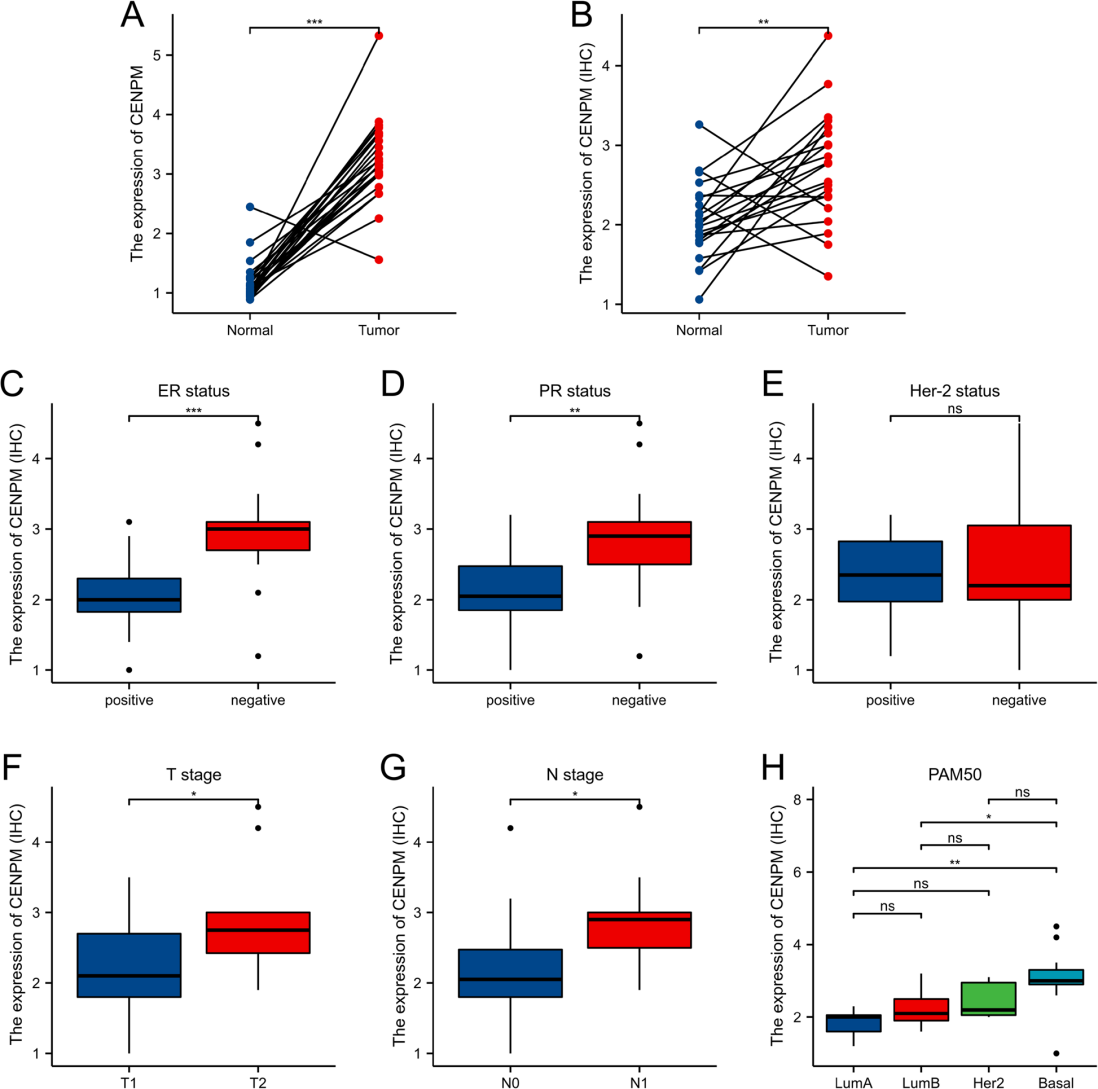
Expression and the relationship between CENPM expression and clinicopathologic features of breast cancer patients in our center. **A** mRNA levels of CENPM in 24 pairs of fresh frozen specimens **B** Protein levels of CENPM in 21 pairs of paraffin sections **C** ER status; **D** PR status; **E** HER-2 status; **F** T stage; **G** N stage; **H** PAM50; *p < 0.05, **p < 0.01, ***p < 0.001

**Fig. 5:**
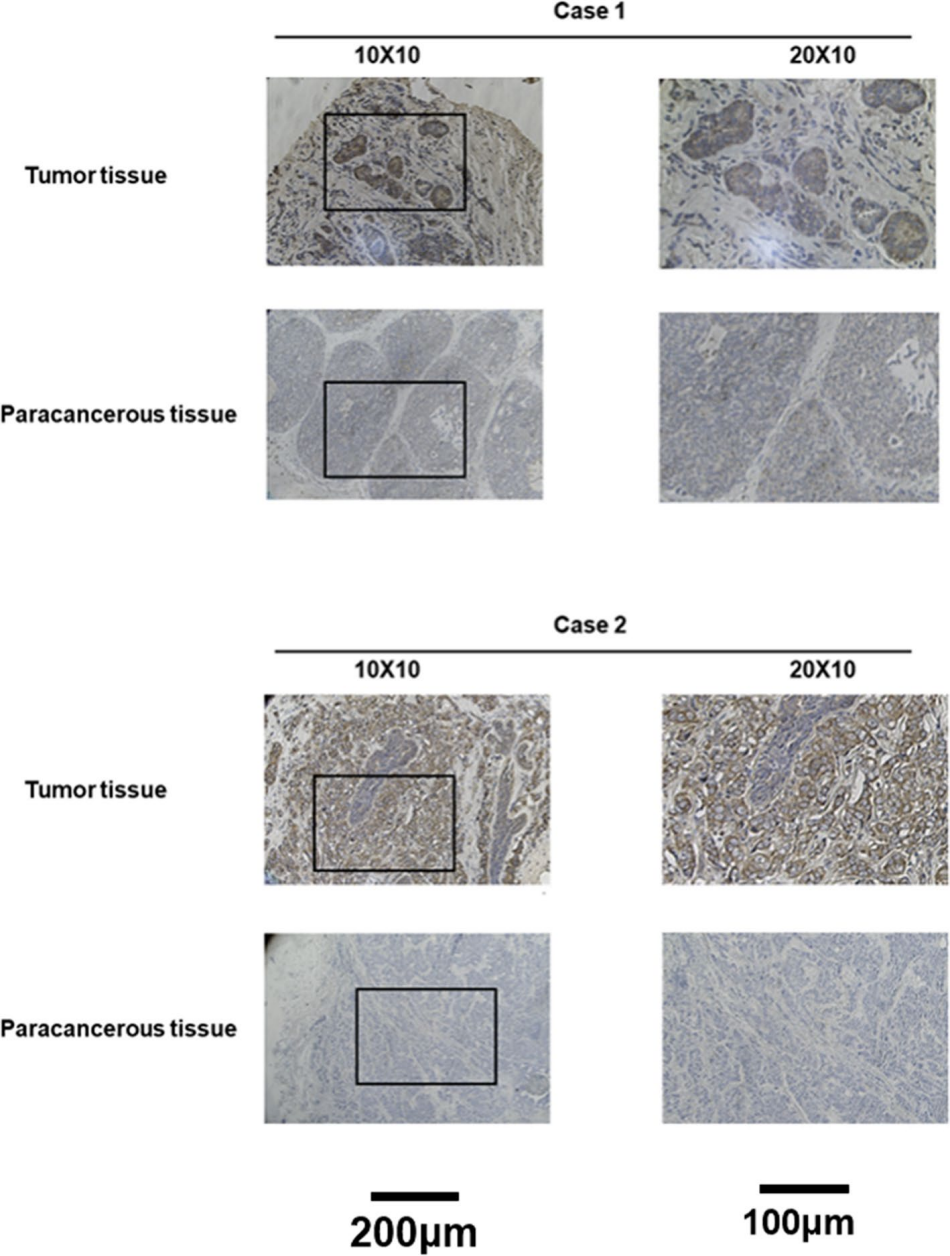
Representative images of CENPM expression in breast cancer tissues and their matched paracancerous tissues. Original magnifications 100 × and 200 × (inset panels)

PAM50 showed significant differences between Luminal B and Luminal A (*p* < 0.001) as well as between Luminal A and Basal (*p* < 0.001). Race also had significant differences, with Asian vs. White (*p* < 0.001) and White vs. Black or African American (*p* < 0.001) (Fig. 3). At our center, we confirmed the overexpression of CENPM in breast cancer samples both at the transcriptional level (Fig. 4A) and the translational level (Fig. 4B). A typical immunohistochemical image is illustrated in Fig. 5. The clinicopathological information of the patients was gathered and subjected to statistical analysis by utilizing immunohistochemistry scores. The analysis indicated that CENPM expression was elevated in patients who were negative for ER and PR, and it was found to have no correlation with HER-2 expression. Shown in Fig. 4C-E. A higher T-stage (Fig. 4F) was associated with the overexpression of CENPM in contrast to the N-stage (Fig. 4G), and triple-negative breast cancer exhibited greater CENPM expression when compared to hormone receptor-positive breast cancer (Fig. 4H). The findings mentioned above were largely in line with the bioinformatics analysis. Statistical methods used were overall test (One-way ANOVA) + multiple hypothesis testing (Tukey HSD post hoc test), data processing: log2(value + 1).

### Analysis of correlation and enrichment

**Fig. 6:**
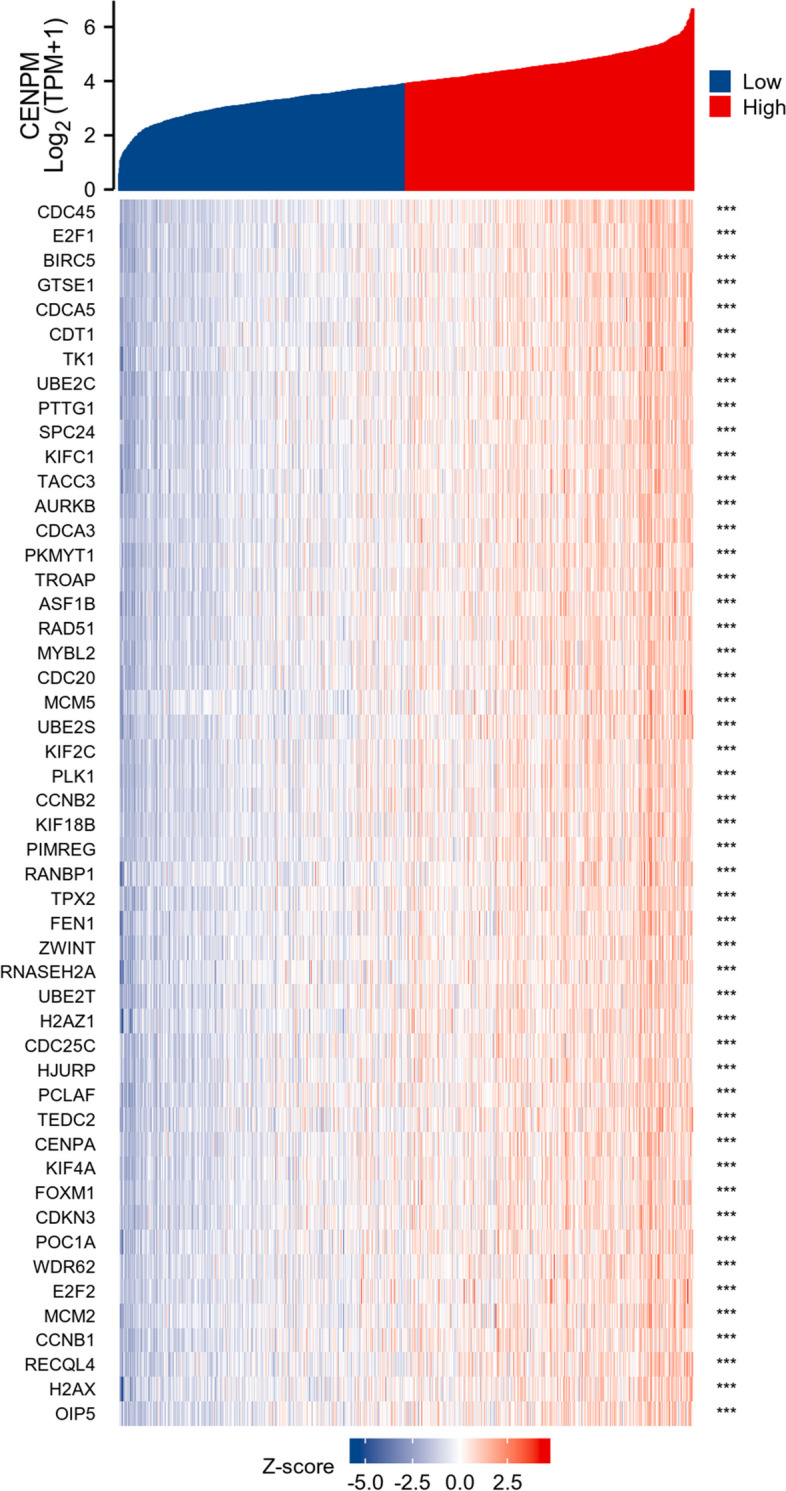
The 50 co-expressed genes with the highest positive correlation of CENPM

**Fig. 7:**
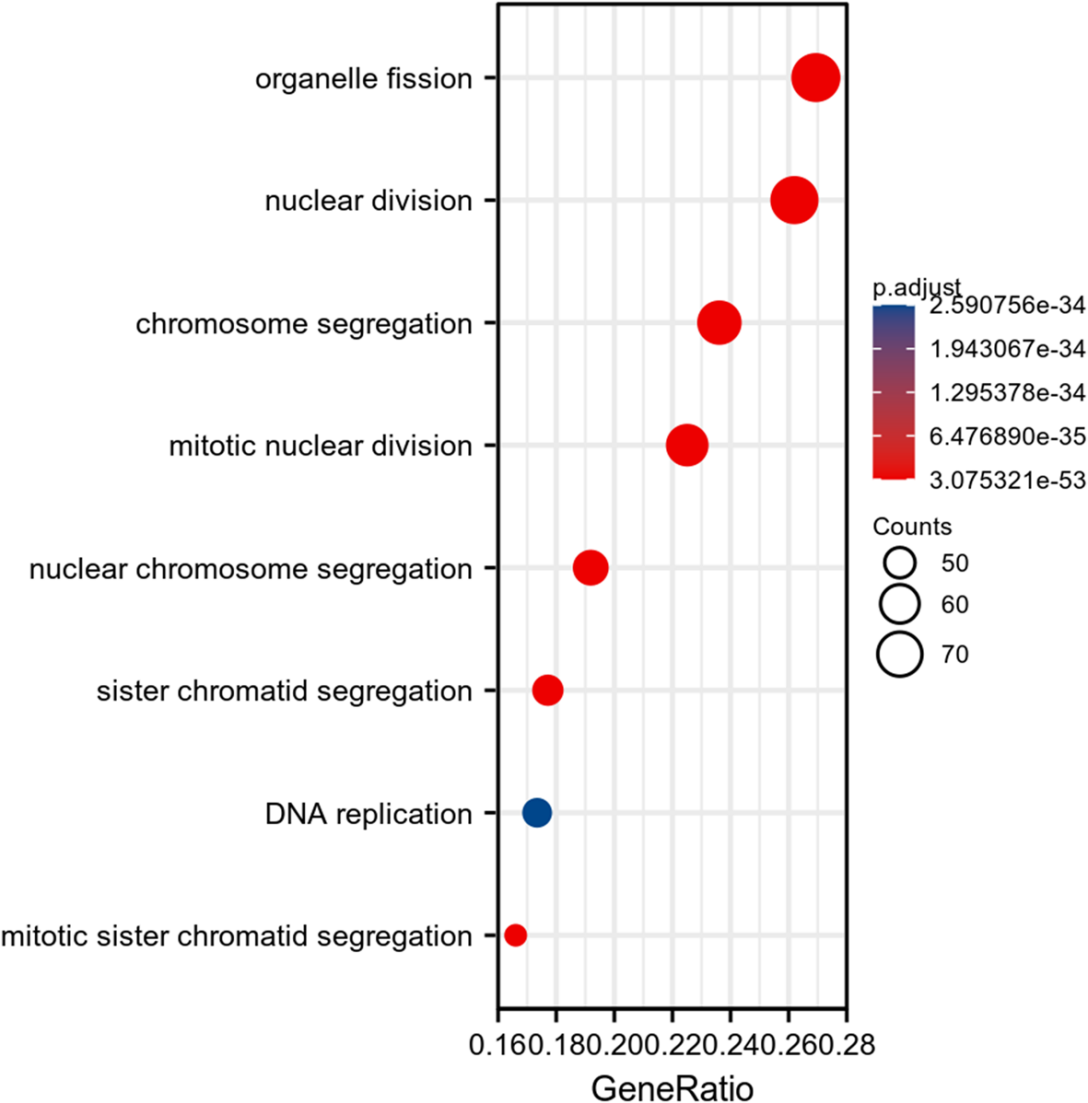
GSEA pathway enrichment analysis of CENPM

**Fig. 8:**
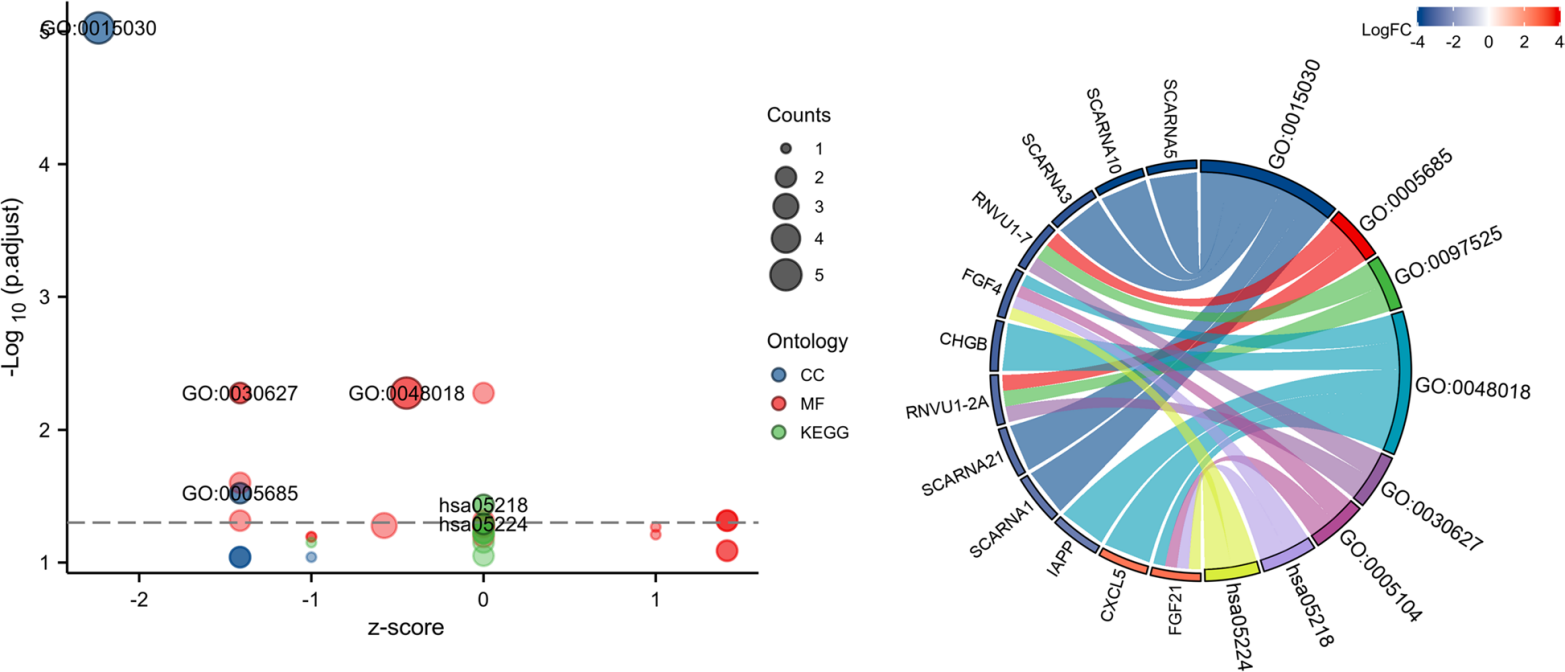
GO/KEGG enrichment analysis of CENPM [17–19]

To investigate the functions and pathways impacted by CENPM, our research analyzed the roles and pathways influenced by CENPM through the utilization of TCGA data. A heat map (Fig. 6) displays the top 50 genes resulting from an enrichment analysis conducted on the 300 genes that have the strongest association with CENPM. The GSEA function enrichment analysis of CENPM and the GO/KEGG pathway enrichment analysis suggest that CENPM has a significant impact on pathways related to cell proliferation, such as nuclear division (Figs. 7 and 8). Elevating the expression of CENPM in breast cancer could potentially lead to the overactivation of various oncogenic pathways, particularly those involved in regulating cell proliferation.

### Infiltration of immune cells and the expression of CENPM

**Fig. 9:**
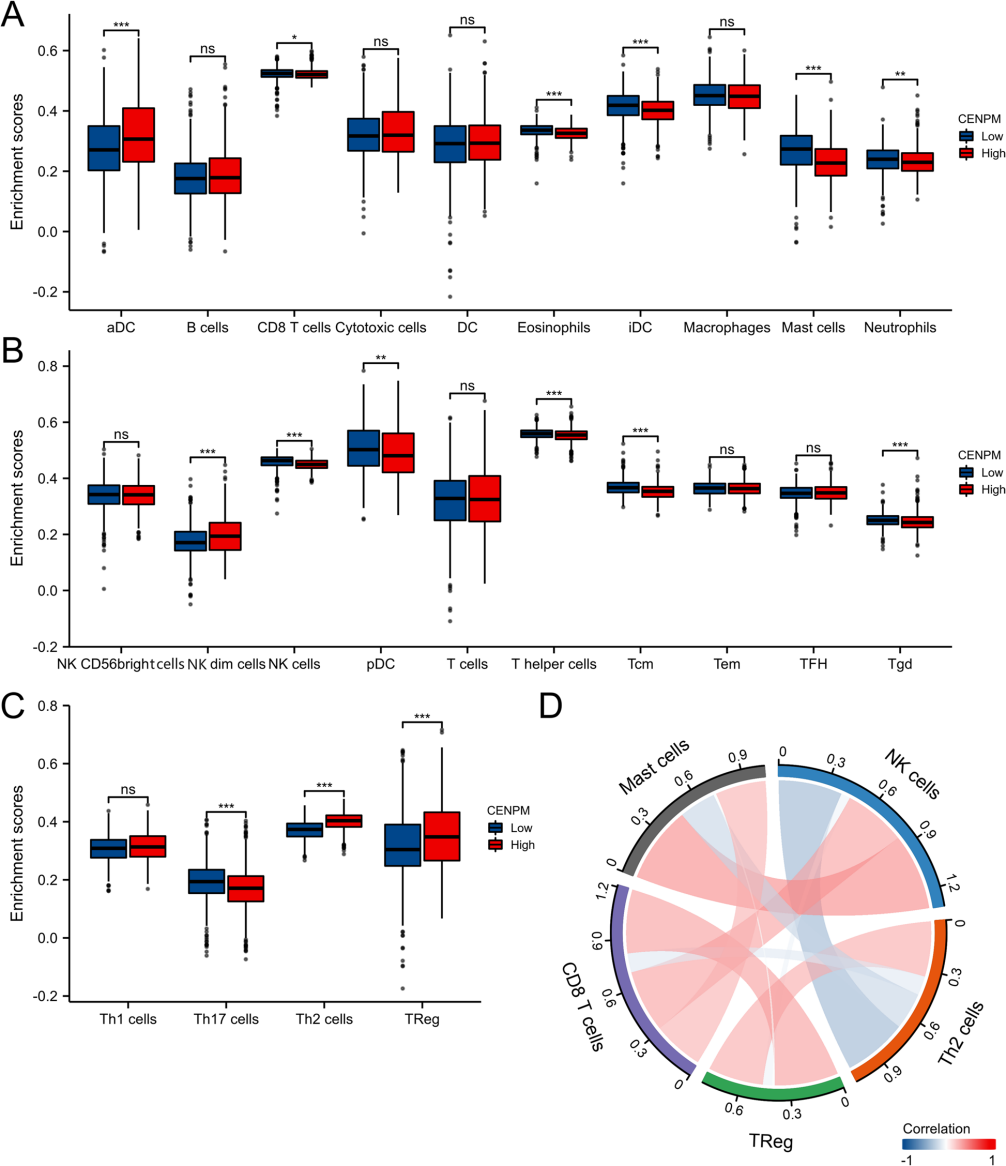
Analysis of CENPM and immune cell infiltration groups. **A**-**C** Grouping of immune cells based on CENPM expression levels. **D** Correlation of 5 immune cells

**Fig. 10:**
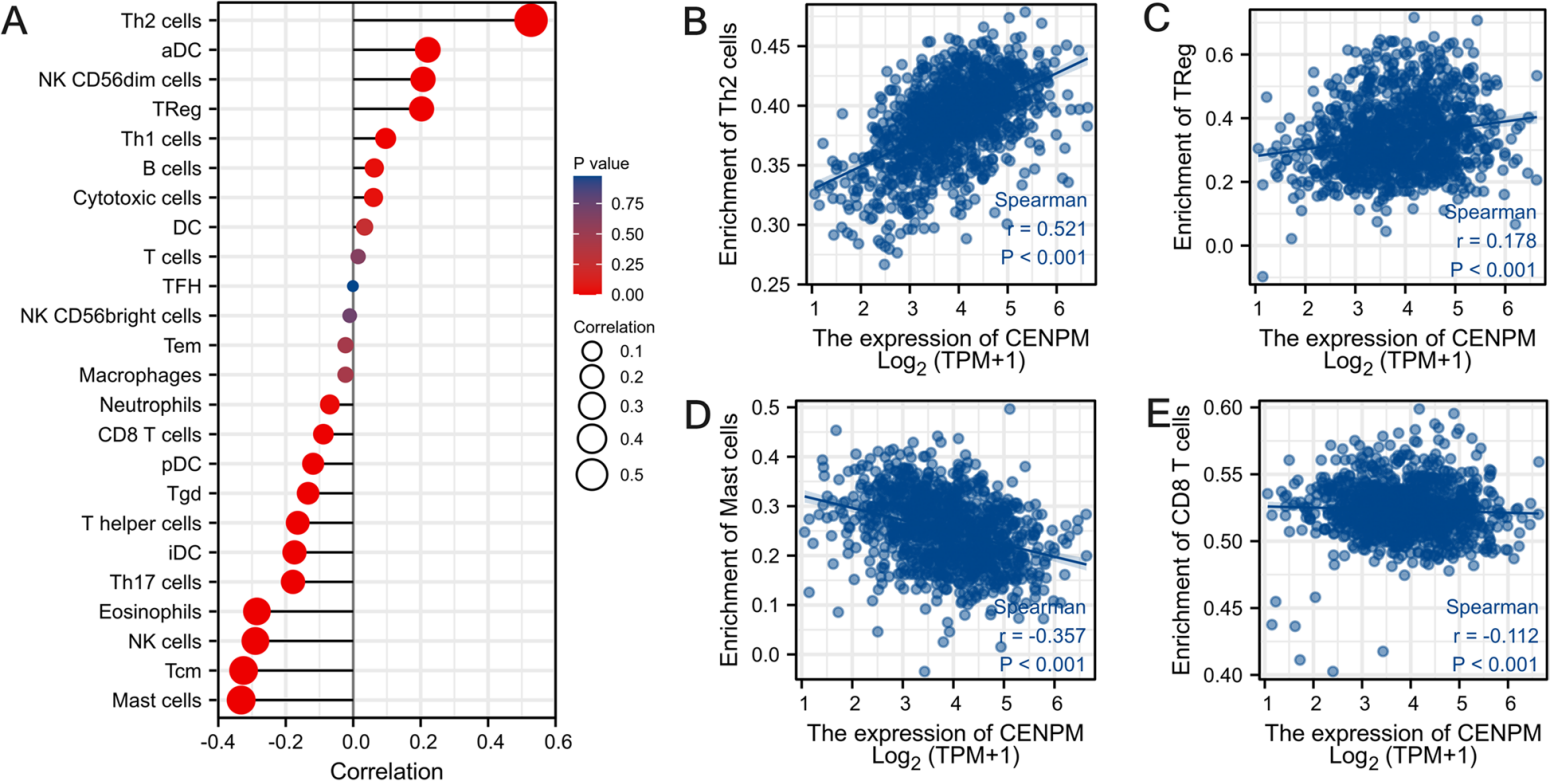
There is an association between CENPM and immune cell infiltration. Infiltration of immune cells has been shown to be correlated with CENPM expression **A**. Infiltration of Th2 cells has been shown to be correlated with CENPM expression (**B**). **C** Correlation between CENPM expression and Treg. **D** Correlation between CENPM expression and Mast cells. **E** correlation between CENPM expression and CD8 + T cells

**Fig. 11:**
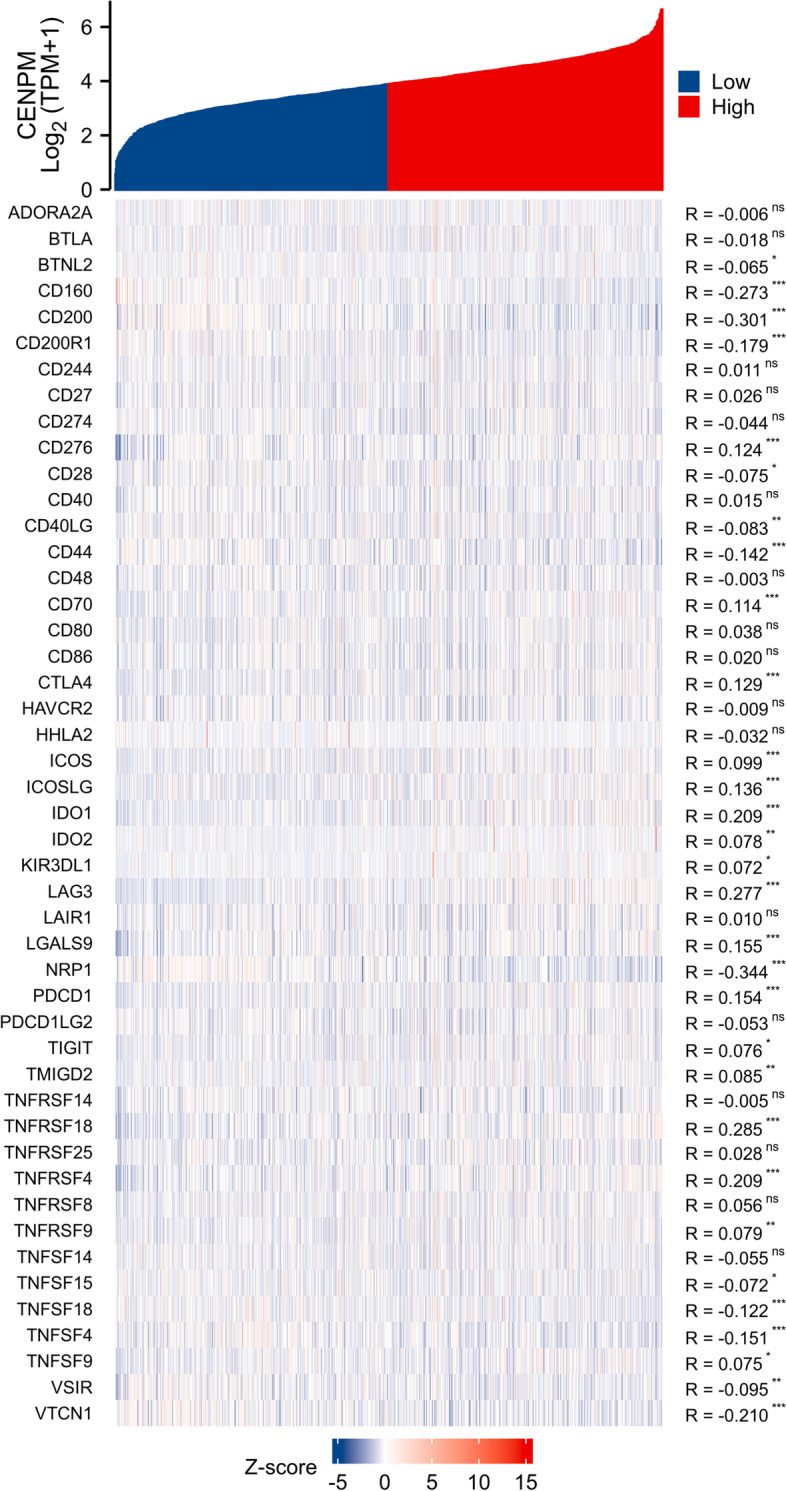
Genes related to immune checkpoints and CENPM co-expressed

**Fig. 12:**
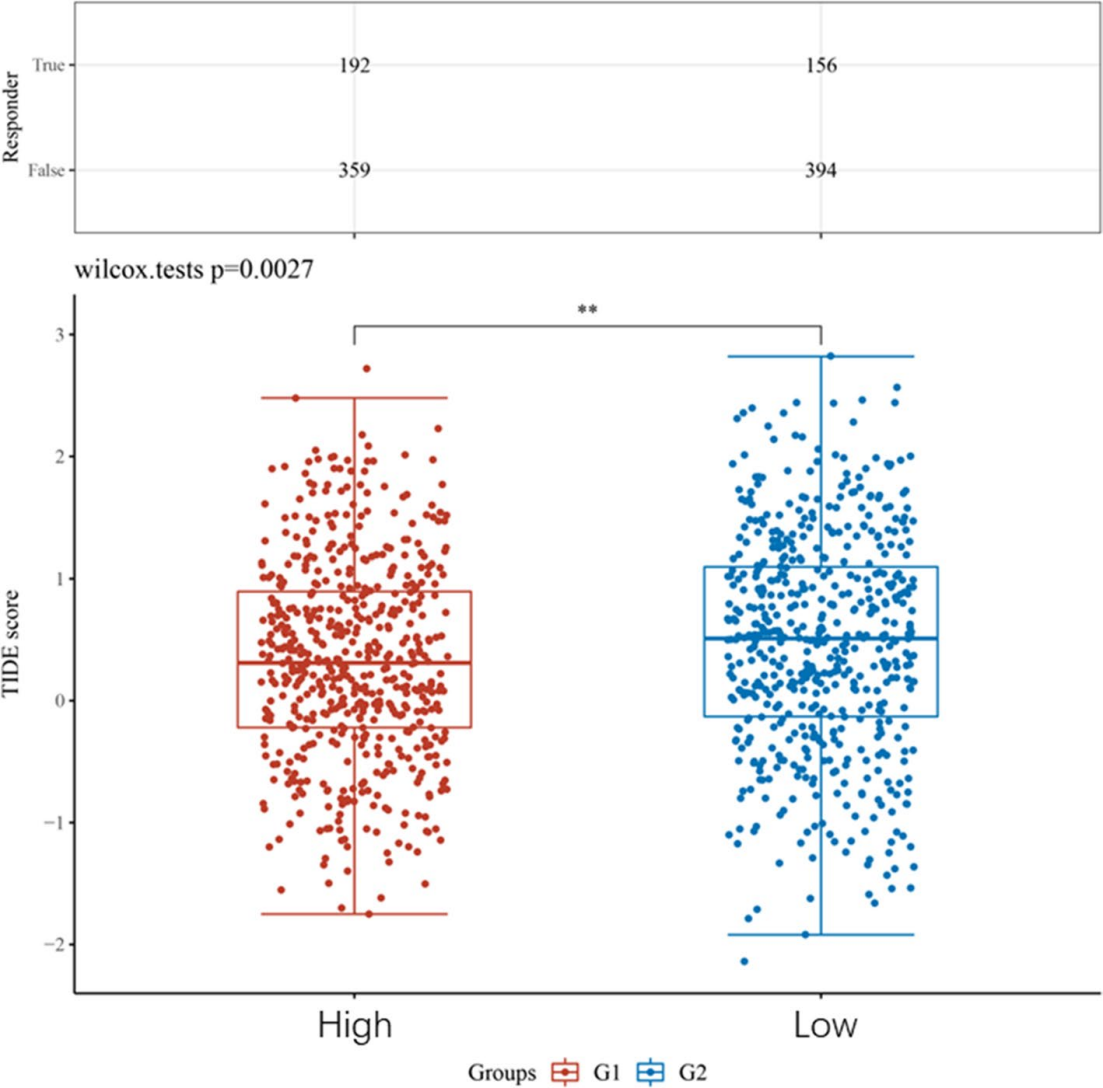
TIDE based on the expression level of CENPM

Afterwards, we examined the TCGA repository for infiltration scores of immune cells in patients with BRCA (Fig. 9). According to Fig. 10, increased levels of CENPM correlated with elevated levels of Tregs and Th2, while showing decreased levels of CD8 + T cells and MSATs. Increased CENPM levels were observed to enhance the accumulation of Tregs and Th2 cells within the tumor, while simultaneously inhibiting CD8 + T cells and Mast cells. The results indicate that an elevated expression of CENPM is associated with the pro-tumor immune condition of breast cancer. Upon investigation of the co-expression of CENPM with genes related to IC in BRCA, it was discovered that CENPM exhibited co-expression with over fifty percent of these genes. The diagram depicted in Fig. 11. In TIDE, groups with high CENPM expression showed greater immunotherapy response [16] (Fig. 12).

**Fig. 13:**
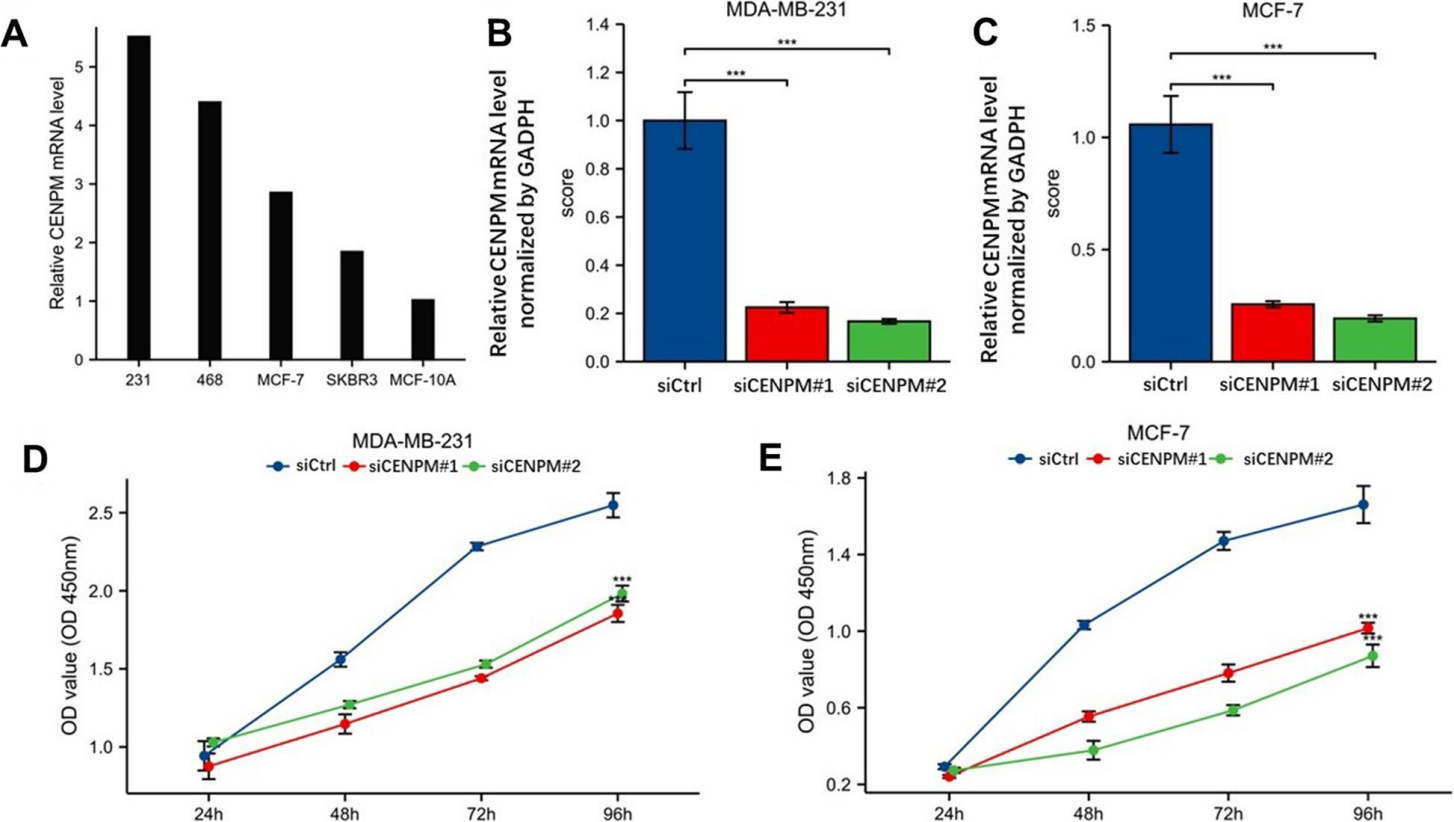
Various cell lines expressed and knocked down CENPM, as well as a proliferation experiment using CCK8 cells. **A** MDA-MB-231, MDA-MB-468, MCF7, SKBRE3, and MCF10A cell lines expressed CENPM. **B** CENPM knockdown efficiency by two siRNAs in MDA-MB-231 cells. **C** Knockdown of two siRNA in MCF7 cell lines Efficiency of CENPM. **D**-**E** Cell proliferation of MDA-MB-231 and MCF7 cells that were knocked down with two siRNA

Suppression of malignant activity in breast cancer cells through CENPM reduction: The expression of CENPM was more pronounced in the four BC cell lines compared to MCF10A.The diagram shown in Fig. 13A. According to Fig. 13B, C, the 231cell line exhibited the highest level of CENPM expression, while the 468cell line showed a lower level.

**Fig. 14:**
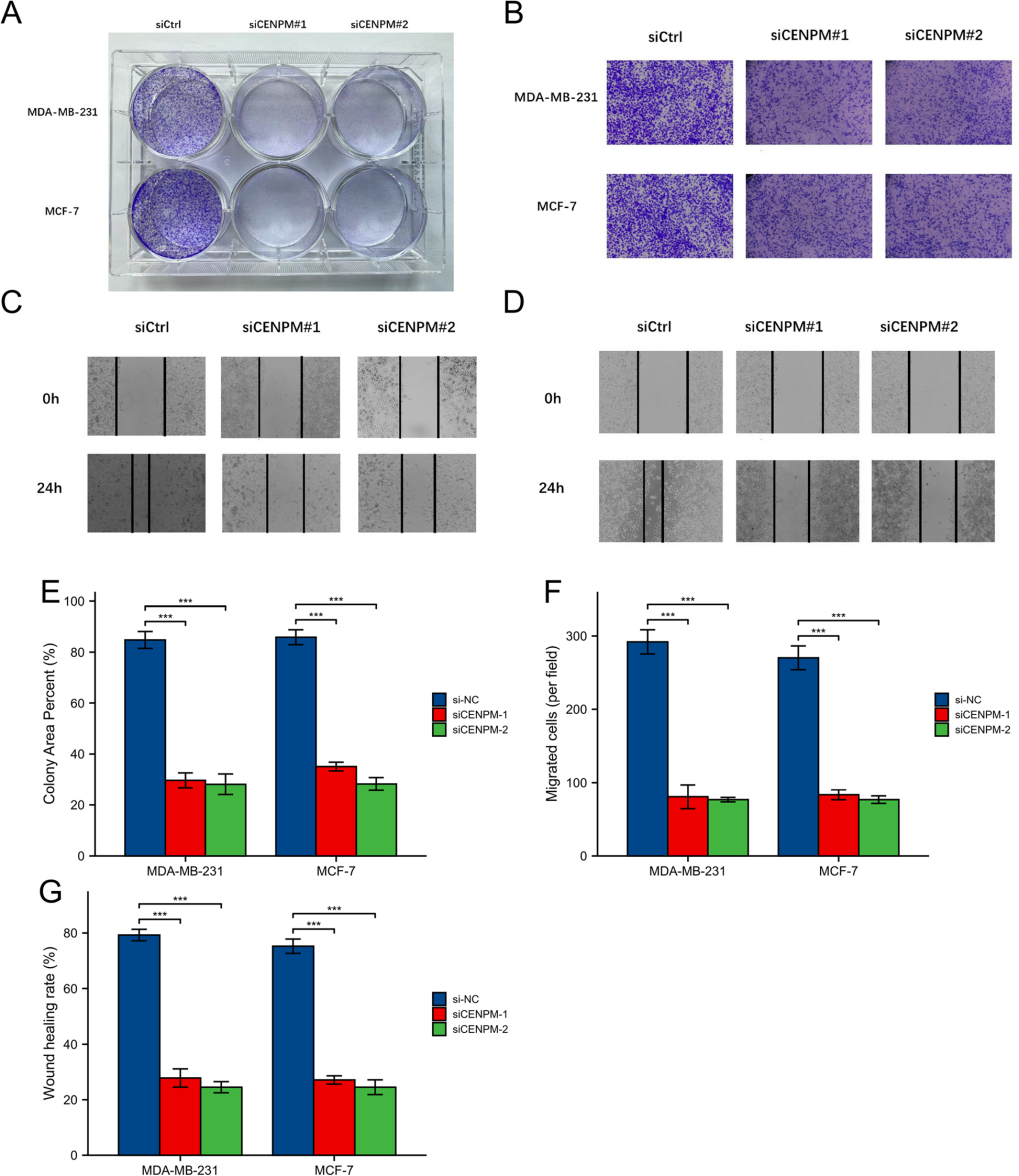
An experiment on colony formation, an experiment on transwells, and an experiment on scratching. **A** Colony formation of control group and two siRNA knockout groups in MDA-MB-231 and MCF7 cell lines. **B** Transwell images of control group and two siRNA knockout groups. The scratch test images of control and siRNA knockout groups in the MDA-MB-231 and MCF7 cell lines are shown in (**C**–**D**). The three-dimensional analysis of colony formation is shown in (**E**). **F** Quantitative analysis of transwell experiment. **G** Quantitative analysis of scratch experiment. All assays were independently repeated at least three times. Data are presented as the mean ± SD ***: *p* < 0.001

**Fig. 15:**
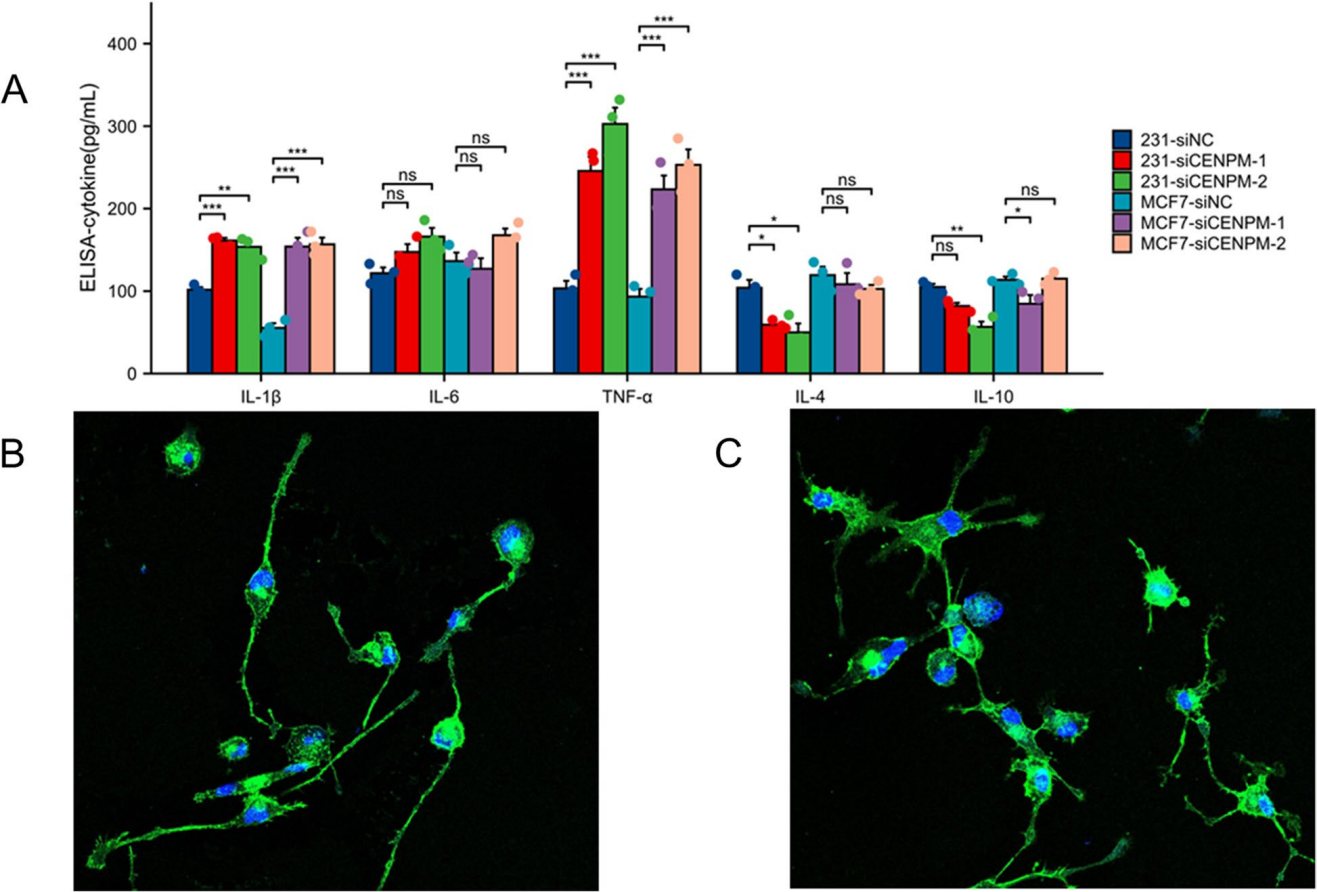
**A** Inflammatory cytokines were measured in the cell supernatants by ELISA. **B** Macrophages treated with siCENPM breast cancer cell supernatants. **C** Macrophages treated with NC breast cancer cell supernatants

In order to validate the impact of CENPM on malignant characteristics of breast cancer, such as proliferation and migration, the CCK8 proliferation assay (Fig. 13D, E) and the colony formation assay were employed (Fig. 14A). The proliferation efficiency of 231 and MCF7 cells with CENPM knockdown was significantly lower when compared to the siCtrl group. Scratch and Transwell assays demonstrated a notable decrease in migration of 231 and MCF7 cells upon CENPM knockdown (Fig. 14B-D).Quantitative and statistical analysis was performed on the results of the colony formation assay, transwell assay, and scratch assay (Fig. 14E-G).To obtain a more comprehensive understanding of how CENPM affects the behavior of breast cancer cells and their immune infiltration, we conducted an experiment where M0 macrophages were cultured with supernatants from breast cancer cells in both the CENPM knockdown and NC groups. Next, we performed ELISA tests to evaluate the occurrence of traditional cytokines released by macrophages. Figure 15A visually illustrates the results of these experiments. The CENPM knockdown cell group exhibited elevated levels of widely recognized pro-inflammatory cytokines, specifically IL-1β and TNF-α, while displaying reduced expression of anti-inflammatory cytokines, particularly IL-4 and IL-10. Confocal microscopy was utilized to analyze the morphology of macrophages(Fig. 15B, C). The polarization of macrophages towards M1 was apparent in the CENPM knockdown group, whereas the NC group showed polarization towards M2.

## Discussion

An increasing population of individuals are facing health hazards due to BRCA, which is currently one of the frequently detected cancers among women. Various treatments, including surgical procedures, chemotherapy, radiation therapy, endocrine therapy, targeted therapy, and more. The treatment strategy encompasses all of these components [20, 21]. However, there are still opportunities for advancements in the management of breast cancer due to the diverse range of tumors and drug resistance. At present, there is no recognized norm for treating this specific disease stage, especially in the case of triple-negative breast cancer [22]. In order to efficiently handle breast cancer, it is essential to conduct screening, identification, and validation of novel causal genes and therapeutic targets. Our study, utilizing bioinformatics and breast cancer samples acquired at our institution, has validated that the overexpression of CENPM is linked to an unfavorable prognosis in breast cancer.

As tumors progress, the levels of CD8 + T cells, NK cells, and other effective lymphocytes that have an anti-tumor function decline, while regulatory T cells and tumor-associated macrophages increase in number [23–25]. The main areas of research to validate the effectiveness of tumor immunotherapy involve enhancing the presence of immune cells that fight against tumors within tumor tissues and inhibiting the growth of cells that promote tumor development. CTLs, which are one of the various immune cell types participating in the tumor immunological process, infiltrate the central region of tumors and function as assassins of cancer cells once they are stimulated. Tregs are characterized by the synthesis of key transcription factors that restrict the proliferation of effector CD4 + T and CTLs. This hinders the tumor host's capability to initiate an effective immune response against the tumor by diminishing immune surveillance [26–29]. When macrophages come into contact with tumor cells in the microenvironment, they have the ability to transform into tumor-associated macrophages [30, 31]. Tumor-associated macrophages (TAMs) have the ability to enhance tumor growth by stimulating tumor angiogenesis, exerting control over persistent inflammation within the tumor microenvironment, and inhibiting immune responses [32, 33]. Based on our analysis, the excessive expression of CENPM results in the infiltration of immune cells that promote tumor growth in breast cancer, while suppressing the infiltration of anti-tumor cells such as CTL and Mast cells. This factor may contribute to the deterioration of prognosis and the advancement of breast cancer in cases of CENPM.

By analyzing the co-expression of CENPM with genes associated with immune checkpoints, we found that over 50% of these genes were co-expressed with CENPM. We compared CENPH(centromere protein H), CENPI(centromere protein I), and CENPK(centromere protein K), which belong to the same CENPs(centromere proteins) family and are closely related to CENPM, which forms the CCAN(constitutive centromere-associated network) network subcomplex with CENPM, but all co-expressed less than 50% of the genes associated with immune checkpoints (CENPH 36%; CENPI 32%; CENPK 42%). This suggests that CENPM might be regulated in conjunction with several targets within the immune checkpoint-associated pathway. By conducting this analysis, we were able to explore the possibility of utilizing CENPM for the clinical management of breast cancer. The TIDE algorithm utilizes a collection of gene expression data. Research has demonstrated that elevated TIDE scores are linked to an inefficient ICB and a brief period of survival after ICB. According to TIDE data, the group with high expression of CENPM exhibited a more robust reaction to immunotherapy. As a result, CENPM has the potential to become a novel target for immune checkpoint inhibitor treatment or a biomarker for predicting effectiveness.

Nevertheless, further investigation is required to ascertain the mechanism behind the involvement of CENPM in the progression of breast cancer. Although statistically significant, the low correlation coefficients of CENPM effects on Treg and CD8 + T cells appear to make it difficult to explain the function of CENPM by promoting cancer-suppressing immunity and suppressing anticancer immunity; thus, the mechanism of CENPM function for tumor immunity needs to be further explored and confirmed. The significance of CENPM's relationship with immune checkpoint inhibitor-related pathways must also be confirmed. Additionally, it is crucial to validate its significance in predicting the effectiveness of breast cancer immunotherapy through comprehensive and prolonged follow-up data.

## Conclusions

Our findings indicate that CENPM may function as an oncogene in breast cancer, as well as a new target for immune checkpoint inhibitors.

## Data Availability

All datasets are freely available from public databases. The study results are mainly based on data obtained from TCGA (https://portal.gdc.cancer.gov/). The datasets used and/or analyzed during the present study are available from the corresponding author on reasonable request.
